# Lipoprotein lipase as a target for obesity/diabetes related cardiovascular disease

**DOI:** 10.3389/jpps.2024.13199

**Published:** 2024-07-16

**Authors:** Rui Shang, Brian Rodrigues

**Affiliations:** ^1^ Lunenfeld-Tanenbaum Research Institute, Mount Sinai Hospital, Toronto, ON, Canada; ^2^ Faculty of Pharmaceutical Sciences, University of British Columbia, Vancouver, BC, Canada

**Keywords:** cardiomyopathy, lipoprotein, atherosclerosis, fatty acid metabolism, cardiomyocytes

## Abstract

Worldwide, the prevalence of obesity and diabetes have increased, with heart disease being their leading cause of death. Traditionally, the management of obesity and diabetes has focused mainly on weight reduction and controlling high blood glucose. Unfortunately, despite these efforts, poor medication management predisposes these patients to heart failure. One instigator for the development of heart failure is how cardiac tissue utilizes different sources of fuel for energy. In this regard, the heart switches from using various substrates, to predominantly using fatty acids (FA). This transformation to using FA as an exclusive source of energy is helpful in the initial stages of the disease. However, over the progression of diabetes this has grave end results. This is because toxic by-products are produced by overuse of FA, which weaken heart function (heart disease). Lipoprotein lipase (LPL) is responsible for regulating FA delivery to the heart, and its function during diabetes has not been completely revealed. In this review, the mechanisms by which LPL regulates fuel utilization by the heart in control conditions and following diabetes will be discussed in an attempt to identify new targets for therapeutic intervention. Currently, as treatment options to directly target diabetic heart disease are scarce, research on LPL may assist in drug development that exclusively targets fuel utilization by the heart and lipid accumulation in macrophages to help delay, prevent, or treat cardiac failure, and provide long-term management of this condition during diabetes.

## Introduction

Continuous beating is a distinctive feature of the heart. As such, cardiomyocytes, which are responsible for this heart contraction, have a high requirement for energy and acquire it from several sources like fatty acids (FA) and glucose in addition to amino acids, lactate and ketones. Among these, the majority of ATP produced in the heart is made from glucose and FA through mitochondrial metabolism, with FA being the favored substrate. The heart is unable to synthesize FA and obtains it from other sources. These include a) release from adipose tissue triglyceride (TG) stores, b) endogenous TG within lipid droplets in the heart, and c) breakdown of circulating TG-rich lipoproteins to FA by lipoprotein lipase (LPL) positioned at the endothelial cell (EC) surface of the coronary lumen. Of these, LPL-mediated breakdown of lipoproteins is suggested to be a major source of FA for cardiac energy generation. This review will cover the participation of LPL in FA delivery to the heart (for generation of energy) and adipose tissue (for storage as TG), and the consequences of its tissue mismanagement following diabetes. Specifically, we will focus on LPL function and dysfunction, and its contribution towards the development of both atherosclerosis and cardiomyopathy. It is hoped that by understanding LPL regulation and modification following diabetes, we can advance the clinical management of diabetic heart disease as it relates to FA metabolism.

## Cardiac lipoprotein lipase—preamble

**FIGURE 1:**
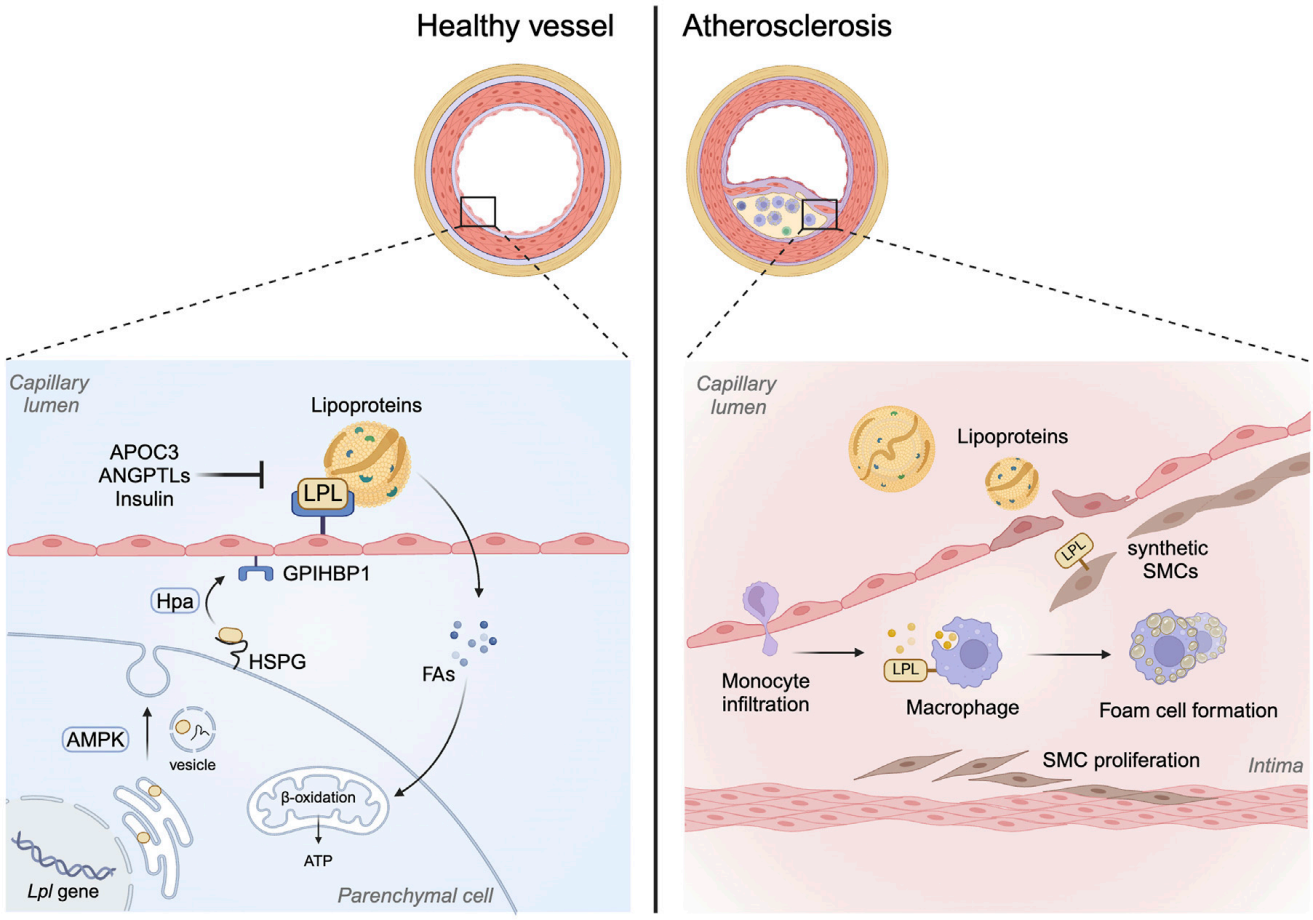
Role of LPL beyond its lipolytic action. LPL has traditionally been known to facilitate lipoprotein hydrolysis to release FA. This action of LPL normally occurs at the endothelial lining of the vasculature following the translocation of LPL from subjacent parenchymal cells to the apical side of endothelial cells. Posttranslationally, LPL activity can be regulated by a number of mechanisms including APOC3, ANGPTLs and insulin. On the other hand, in macrophages, the action of LPL is mainly to promote remnant cholesterol uptake, foam cell formation and plaque development in arteries. This bridging function of LPL in SMCs, especially synthetic cells that make up the plaque, may also contribute towards lipoprotein uptake and foam cell formation.

The breakdown of circulating TG in lipoproteins by LPL occurs in the vascular lumen. However, endothelial cells (EC) that line the lumen are incapable of producing LPL [1–3]. Using the heart (where LPL can be examined at different sites), it has been documented that this enzyme is made in cardiomyocytes before it is moved to the coronary lumen. Thus, immunogold labeling of LPL confirmed that in the heart, about 80% of LPL is present in cardiomyocytes, 18% is located at the capillary EC, and the remaining amount is located in the interstitial space (Figure 1) [4]. Related to its synthesis in cardiomyocytes, LPL has been reported to be produced as a monomer (inactive) in the endoplasmic reticulum. Enzyme activation follows dimerization, with subsequent cellular secretion [5, 6]. Recent evidence has suggested that monomeric LPL also shows enzyme activity [7]. Following its synthesis, there are two proteins that are important for LPL maturation (folding) and transport; lipase maturation factor and suppressor of lin-12-like protein 1 [8]. Intracellular vesicles then store LPL bound to syndecan-1 [9]. Vesicular movement to the cell surface permits LPL secretion onto heparan sulphate proteoglycan (HSPG) binding sites on the outer surface of cardiomyocytes [10]. Attachment of the positively charged LPL to HSPG is made possible by ionic binding to the heparan sulfate (HS) side chains that is negatively charged. Location of LPL at this site serves as a rapidly accessible pool for the heart when it requires energy in the form of FAs [11, 12]. For its onward movement from the myocyte cell surface to the coronary lumen, LPL is released from HSPG, crosses the interstitial space and binds to glycosylphosphatidylinositol-anchored high-density lipoprotein-binding protein 1 (GPIHBP1) on the basolateral side of EC [13]. GPIHBP1 functions as a transporter, moving LPL from the basolateral side of EC to the capillary lumen [14–16]. At the lumen, GPIHBP1 can bind LPL and circulating lipoproteins to promote TG hydrolysis and supply FA to the underlying cardiomyocytes for energy production [16–18] (see detailed review [19]). Interestingly, the action of LPL on chylomicron (lipoprotein produced mainly in the gut) clearance is likely more substantial than its effect on VLDL (lipoprotein synthesised mainly in the liver) hydrolysis. Compared to VLDL, chylomicrons are larger, contain a greater amount of TG and have a better chance to interact with coronary LPL [20]. In addition, LPL breakdown of VLDL yields FA that require a FA transporter, CD36, for movement into cells, whereas its breakdown of chylomicrons produces a higher amount of FA that use a passive flip-flop and/or CD36-independent transport mechanisms [21]. A more recent study produced lipoproteins by administering radiolabelled ^2^H-FA by gastric gavage. Following isolation and *i*.*v*. injection of the lipoproteins (*d* = 1.006 g/mL), a rapid (as quickly as 2 min) accumulation of radiolabelled FA within the cytosol and mitochondria of cardiomyocytes was observed, indicating that EC did not serve as a storehouse for FA as they traveled from the vascular lumen to the underlying cardiomyocytes [22]. Additionally, as a deficiency in CD36 did not modify the passage of lipoprotein FA into cardiomyocytes, the authors concluded that LPL derived FA can be taken up quickly by cardiomyocytes without the need for FA carriers [22]. Finally, when LPL-derived FA are compared to non-esterified fatty acid (NEFA) derived from adipose tissue, our data suggest that following fasting, NEFA plays a central role in energy generation [23], whereas LPL action also provides for lipid accumulation in the heart [24].

## Posttranslational processes that regulate cardiac LPL

Of the multiple substrates that the heart can use as an energy source, FA is the preferred fuel. As such, intrinsic mechanisms have been developed by the heart to regulate delivery of this substrate, with LPL being a major player. Several mediators are present which modulate cardiac LPL. These include:

### AMP-activated protein kinase (AMPK)

Upon a reduction in energy, AMPK is activated to stimulate energy producing pathways and turn off energy consuming pathways to restore the ATP/ADP ratio. Thus, AMPK is known to inhibit acetyl-CoA carboxylase, lower malonyl-CoA and increase the activity of carnitine palmitoyltransferase-1 to facilitate FA uptake and oxidation in the mitochondria [25, 26]. Stimulation of AMPK is also known to modulate FA uptake via CD36 [27]. Our laboratory has extensively published on the role of AMPK in LPL translocation to the vascular lumen [28]. Specifically, we reported that AMPK plays a role in vesicular formation and subsequent movement of LPL along the actin cytoskeleton in cardiomyocyte through activation of heat shock protein 25 [2]. Thus, physiological and pathological processes that change AMPK are known to impact coronary LPL activity. For instance, following overnight fasting, AMPK is activated to increase coronary LPL, that guarantees FA delivery to meet the energy demand in this nutrient deficient condition [29]. Using streptozotocin (STZ) to induce moderate diabetes in rats, we reported that like fasting, acute hypoinsulinemia stimulated AMPK phosphorylation, and resulted in an augmented coronary LPL activity. This enabled the heart to switch its substrate utilization to exclusively using LPL-derived FA [10]. With a higher dose of STZ to induce severe diabetes, these animals developed both hyperglycemia and severe hyperlipidemia with increased circulating FA [30], that are known to inhibit AMPK activation [31]. In hearts from these animals, LPL activity was reduced and an unregulated uptake of NEFA resulted in cardiac lipotoxicity and dysfunction [30, 32]. Overall, our data suggested that activation of AMPK is a significant contributor towards LPL movement and subsequent FA utilization. Thus, agents that are capable of increasing cardiac LPL activity through the AMPK pathway may be useful for preventing NEFA uptake and lipotoxicity following diabetes.

### Heparanase (Hpa)

Hpa is an endo-β-glucuronidase that is produced in EC as an inactive latent protein (Hpa^L^). Following its synthesis, it is secreted to be taken up by HSPG and stored in lysosomes [33, 34]. At this location, enzyme processing results in a 50-kDa polypeptide that is significantly more active (Hpa^A^) than Hpa^L^ [35, 36]. Both forms of Hpa are stored within the EC until secreted in response to various stimuli. Related to its physiological functioning, Hpa has roles in embryonic development, wound healing and hair growth [37]. Studies from our lab was the first to identify a novel role of Hpa in cardiac metabolism. In this regard, we described how Hpa released cardiomyocyte LPL for subsequent transfer to the vascular lumen for FA generation [38]. In people living with Type 2 diabetes, plasma and urine levels of Hpa are increased [39, 40]. *In vitro* studies using EC established that acute incubation of these cells with high glucose had a robust influence on Hpa secretion [41]. Using an animal model of STZ-induced diabetes, isolated hearts released significantly higher amounts of both forms of Hpa within the first 5 min, with Hpa^L^ secretion being greater than Hpa^A^ [42]. Related to Hpa^A^, its heparan sulfate hydrolyzing ability would be capable of releasing myocyte surface-bound proteins including LPL. Intriguingly, enzymatically inactive Hpa^L^ is also able to initiate HSPG-clustering that activates p38 MAPK, Src, PI3K-Akt, and RhoA [38, 43–46]. These signalling pathways could then allow for replenishment of the cardiomyocyte pool of LPL that was released by Hpa^A^. Overall, circulating Hpa has an important role in the communication between EC and cardiomyocytes to eventually supply FA to the heart. Intriguingly, unlike high glucose, when EC are exposed to increasing concentrations of palmitic acid, the nuclear content of Hpa was augmented [41]. Moreover, in recently published data from our lab, severe diabetes with concomitant hyperglycemia and hyperlipidemia reduced Hpa secretion from the isolated heart, a possible explanation for the lowered coronary LPL activity in these hearts [47]. Currently, whether manipulation of EC Hpa is capable of influencing cardiac metabolism following diabetes is unknown and should be investigated.

### Heparan sulfate proteoglycans (HSPG)

To determine the contribution of HSPG to LPL transcytosis, LPL accumulation was determined following knock-out of GIPHBP1. In this condition, LPL in skeletal muscle and heart collected more at the basolateral side of EC as compared to the cardiomyocyte side suggesting that an HSPG gradient determines the direction of LPL flow from the underlying cardiomyocyte to the basolateral surface of EC [48]. At this location, collagen XVIII also acts as a reservoir for LPL [49].

### GPIHBP1

LPL is expressed mainly in parenchymal cells like cardiomyocytes, whereas GPIHBP1 is located exclusively in capillary ECs. Interestingly, a comparable distribution of GPIHBP1 is described at both the luminal or abluminal sides of these cells [16, 50]. It should be noted that ECs that are part of large blood vessels, arterioles and venules do not display GPIHBP1 [16]. Regarding the binding of LPL to GPIHBP1, this occurs at a 1:1 ratio and with a higher affinity when compared to its binding to HSPG [51]. Structurally, as GPIHBP1 contains a GPI anchor, its release from the plasma membrane is achievable with phosphatidylinositol-specific phospholipase C that is known to digest this anchor [14]. It is the acidic domain of GPIHBP1 that can ionically attach LPL [52]. Given its defined role in the bidirectional translocation of LPL across the EC [16], and its ability to serve as a platform to promote lipoprotein-TG hydrolysis (it allows lipoproteins to stay bound/marginate to heart capillaries for several minutes [53, 54]), its absence in GPIHBP1 knockout mice causes robust hypertriglyceridemia even when these animals are fed a low-fat diet [16]. Similar effects are seen in patients with GPIHBP1 mutations [55]. More recent functions of GPIHBP1 include its ability to prevent the unfolding of LPL by angiopoietin-like protein 4 (ANGPTL4) [56–58]. Regarding its regulation, GPIHBP1 expression can be affected by fasting/refeeding [59]. Fasting augments cardiac GPIHBP1, and this effect can be overcome by refeeding [59]. Following diabetes, cardiac GPIHBP1 gene and protein expression also increase with an associated augmentation of coronary LPL activity [60]. Moreover, *in vitro* incubation of EC with high glucose also caused a rapid increase in GPIHBP1 mRNA and protein [50, 61]. Intriguingly, exposure of EC to Hpa^L^ or Hpa^A^ produced a significant increase in GPIHBP1 gene and protein [50]. Given that high glucose can stimulate the secretion of both forms of Hpa, this could be one mechanism by which the EC can increase GPIHBP1 to accelerate FA delivery to the cardiomyocytes after diabetes.

### Angiopoietin-like proteins (ANGPTLs) regulation of LPL

ANGPTL 3, 4, and 8 are endogenous LPL antagonists. ANGPTL3 is exclusively expressed in the liver whereas ANGPTL4 and 8 are abundant in the liver, adipose tissue and muscle. One way by which fasting decreases LPL in the adipose tissue is that nutritional deprivation increases ANGPTL4 in adipose tissue. This is a positive effect as circulating TG are then diverted towards oxidative tissues for provision of energy [62].

### Fatty acids (FA)

FA is known to affect LPL in multiple ways, including a) FA inhibition of LPL movement in the cardiomyocyte [63], b) FA suppression of Hpa secretion, thus reducing cardiomyocyte to EC transfer of LPL [41], c) FA detachment of vascular LPL for hepatic degradation [64], and d) FA inactivation of LPL, either directly [65] or through induction of ANGPTL4 [66–68]. With severe diabetes, animals developed hyperlipidemia that was associated with a reduction in heparin-releasable LPL activity in the heart [32]. This occurred in the absence of any change in LPL gene expression [69] suggesting that following diabetes, cardiac LPL activity is mainly modulated by post-translational mechanisms. In this regard, when RNA-seq was performed in diabetic hearts, of the more than fifteen hundred differentially expressed genes, the one that showed the greatest fold change (∼25-fold increase) was ANGPTL4 [30]. Altogether, these results imply that circulating FA has the ability to supress vascular LPL by a host of mechanisms to prevent lipid overload of the heart.

### Insulin

Changes in circulating insulin can affect LPL and this response varies with the tissues being studied [70]. Thus, a reduction in insulin after fasting decreases adipocyte LPL but enhances its activity in the heart [71], changes that occurred in the absence of LPL gene or total protein expression [29]. Consequently, the FA that are produced from lipoprotein-TG lipolysis by LPL are directed away from storage in the adipose tissue so that they can fulfill the metabolic demands of cardiomyocytes. As newly synthesized LPL can transfer from myocytes to the vascular EC within 30 min, an augmented vectorial movement of LPL could explain the rapid increase of coronary LPL following fasting [72]. Mechanistically, a reduction in insulin after fasting or STZ-induced diabetes decreases glucose uptake in the heart resulting in activation of AMPK [1, 73, 74] with stimulation of LPL translocation [2] (see detailed review [19]).

### Apolipoproteins

Activators of LPL include Apolipoproteins (Apo) C-II and Apo A-V, whereas inhibitors include Apo C-III. Apo-CII is produced primarily in the liver and then incorporated into lipoproteins. On binding to LPL, it promotes conformational changes in the enzyme, allowing the catalytic site of LPL to interact with lipoproteins permitting their hydrolysis [75]. Apo A-V increases the activity of Apo C-II [76, 77].

## Oscillations in cardiac LPL following diabetes and its impact on plasma triglycerides

In the clinical setting, plasma LPL activity is determined after infusion of heparin to release HSPG-bound LPL [12, 78]. The downside with this method is that the measured LPL represents enzyme that is released from a host of different tissues (heart, skeletal muscle, adipose tissue). Regarding tissue-specific detection of LPL following diabetes, adipose tissue and skeletal muscle show low levels of enzyme in homogenates [79], with virtually no information available on the cardiac content of this enzyme. Even if heart homogenates are used to determine LPL levels, this would only provide an estimation of total cardiac LPL and would not correctly reflect the enzymatically active LPL at the vascular lumen. Hence, studies in animals have provided the key source of information regarding LPL biology in the diabetic heart. Thus, acute insulin resistance following administration of dexamethasone [80, 81] or hyperglycemia and hypoinsulinemia in rats injected with 55 mg/kg STZ (D55) causes a significant increase of heparin-releasable LPL at the coronary lumen [10, 32, 82, 83]. This increase occurred due to a rapid filling of the unoccupied HSPG-binding sites [70, 82, 84] and was independent of changes in LPL gene and protein expression [82, 85]. Occupation of these empty HSPG-binding sites at the EC surface was mediated by enhanced translocation of LPL [29, 38, 41, 63, 69, 70, 86–91].

For a considerable period, it has been undecided whether the hypertriglyceridemia following diabetes was an outcome of augmented synthesis of VLDL-TG from the liver or a product of reduced clearance of lipoprotein-TG by LPL. In the moderately diabetic D55 animals with 2-fold increase in cardiac LPL activity, circulating TG and NEFA were maintained at levels that were comparable to control animals [30]. Willecke et al. using STZ-diabetic mice revealed that VLDL secretion remained unchanged in these mice [92]. Interestingly however, TG clearance was significantly reduced and was related to a reduction in skeletal muscle, cardiac, and brown adipose tissue LPL mRNA/activity, suggesting that LPL clearance of TG is the more important contributor.

**FIGURE 2:**
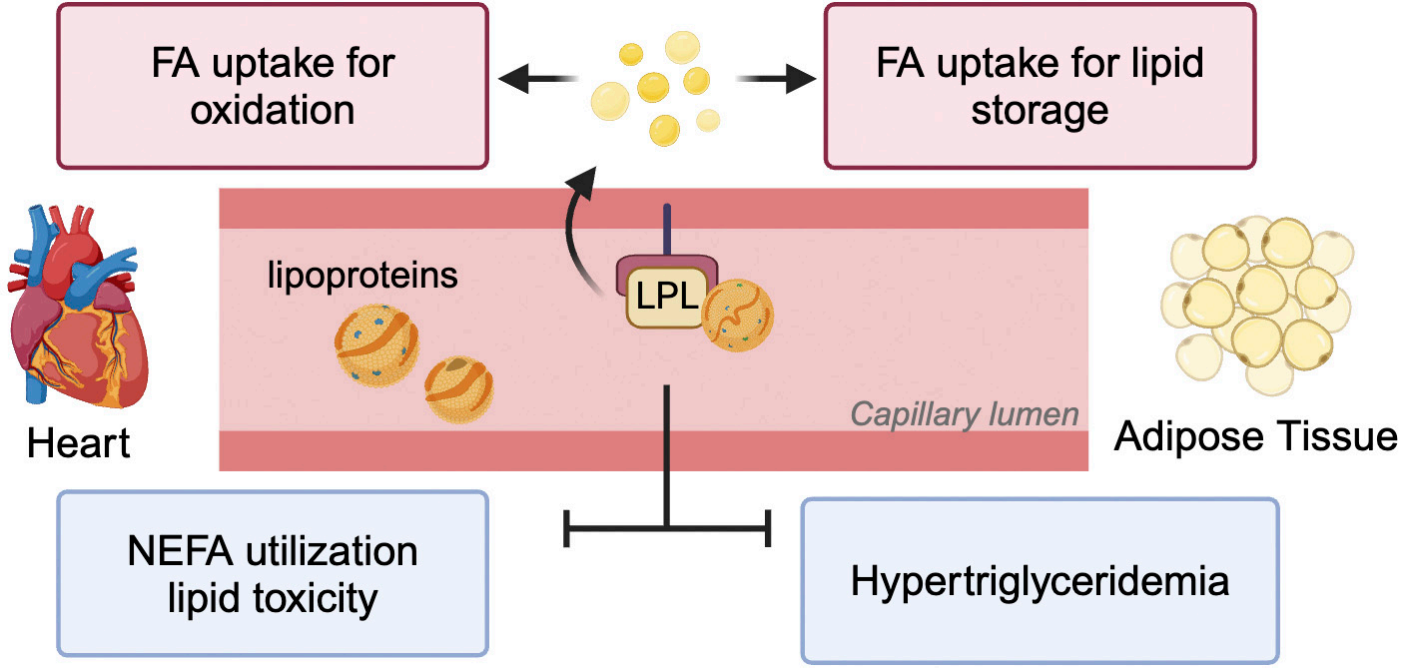
Impact of LPL-derived FA on heart and adipose tissue. LPL positioned at the vascular lumen is responsible for lipoprotein-TG break down to release FA for multiple purposes. These include uptake into the heart for generation of energy and adipose tissue for lipid storage. In pathological conditions like diabetes when LPL activity is reduced in both these tissues, the reduction in TG clearance results in hypertriglyceridemia. Additionally, the heart switches to using NEFA for oxidation leading to excessive TG accumulation and lipotoxicity.

In diabetes, an increase in circulating and intracellular TG are risk factors associated with atherosclerotic cardiovascular disease and cardiac lipotoxicity [93]. It is possible that in D55 heart, LPL derived FA are directed towards oxidative metabolism rather than storage (Figure 2) [30]. Intriguingly, similar to our observations in the moderately diabetic D55 heart, a modest overexpression of LPL in adipose tissue was associated with better glucose and insulin tolerance [94]. When these animals were provided a high fat diet, weight gain was not observed. In fact, dietary lipids did not accumulate in adipose tissue, and the animals displayed amplified energy expenditure. The authors proposed that a moderate increase in adipose LPL has favourable effects on total body energy metabolism. In contrast, we also observed a decline in LPL, both in animals infused with Intralipid [95] and with severe diabetes induced by injecting 100 mg/kg STZ (D100) [10, 30]. As the D100 diabetic animals exhibit elevated plasma FA, we concluded that LPL-mediated FA delivery would be redundant in these circumstances and is “turned off.” Additionally, this reduced cardiac LPL likely contributed to the robust increase in circulating TG. We have previously shown that when FA uptake by LPL action is augmented, this competes with NEFA uptake [23]. Thus, following severe diabetes, when cardiac LPL action is reduced, NEFA uptake and oxidation takes precedence over provision of FAs to the heart from circulating lipoproteins. This excessive supply of NEFA overwhelmed the mitochondrial capacity, leading to a mismatch between FA delivery and utilization, lipid metabolite build-up and cell death (Figure 2) [30]. Thus, approaches that maintain cardiac LPL would be a useful therapeutic approach to preventing cardiac pathology seen following diabetes that is poorly controlled.

## Role of LPL in development of atherosclerosis

Atherosclerosis is defined as a thickening (and loss of elasticity) of the arterial intima as a consequence of lipid accumulation [96]. During this condition, there is narrowing or obstruction of the vessel lumen and thinning of the vessel wall. In preclinical animal models, atherosclerosis is a progressive disease beginning with the development of fatty streaks and potentially leading to complicated atherosclerotic plaques that can rupture, set up thrombosis and occlude the lumen. The clinical manifestations of atherosclerosis are dependent on the site of lesion. Hence, its presence at the coronary arteries leads to angina pectoris and myocardial infarction, at the central nervous system it causes transient cerebral ischemia and stroke whereas its occurrence in the peripheral circulation elicits peripheral vascular disease [96]. The level of plasma cholesterol, and in particular LDL-associated cholesterol, is one of the main risk factors of atherosclerosis [97]. Following LDL infiltration and trapping in the arterial intima with potential oxidative modification, ox-LDL causes endothelial cells to express monocyte chemoattractant protein (MCP-1). MCP-1 attracts monocytes from the vessel lumen into the subendothelial space, one of the very early stages in the development of atherosclerosis. Modified LDL also promotes differentiation of monocytes into macrophages which avidly take up the ox-LDL. This accumulation transforms macrophages into lipid-rich foam cells, that are the hallmark of atherosclerosis [96]. Engorgement of foam cells with lipids causes release of cytokines, and eventually cell death. Macrophage/foam cell-released proteolytic enzymes (matrix metalloproteinases, MMPs) allows for smooth muscle cells from the adjacent media to migrate into the intima, proliferate and secrete fibrous connective tissue (i.e., collagen) and extracellular matrix (smooth muscle cells change their phenotype from contractile to synthetic cells). This makes the lesion harder and contributes to the formation of a fibrous cap (which includes a mixture of macrophages, lipid and cell debris which form a necrotic core). The expanding intima pushes against the endothelial wall of the intima and the fibrous cap is very susceptible to rupture. MMPs also cause a thinning of the fibrous cap with eventual cap destruction along with a host of other events like platelet aggregation and adhesion, thrombosis and clot formation. The rupture of such lesions is believed to be responsible for most cases of unstable angina and acute myocardial infarction. Dislodging of the clot blocks the artery near the plaque or in a more distal and narrower segment causing total or near total occlusion [96, 98].

### LPL in macrophages

Given the contribution of LPL in supplying FAs to various tissues for storage and energy generation, in addition to its role in plasma lipoprotein clearance, the action of LPL is considered beneficial. Thus, the contribution of LPL towards the etiology of atherosclerosis is contentious. On the one hand, overexpression of LPL has been shown to protect against diet-induced atherosclerosis in Ldlr^−/−^ and Apoe^−/−^ mice, established animal models to study atherosclerosis [99, 100]. This protective effect of LPL was linked to beneficial changes in plasma lipoproteins. On the other hand, this was not the case when LPL levels were manipulated in macrophages. Specific deletion of macrophage LPL (with no changes in total plasma LPL activity) significantly reduced atherosclerotic lesions in Apoe^−/−^ mice, supporting an important role for macrophage LPL in atherosclerosis [101]. Similarly, overexpression of human LPL in rabbit macrophages accelerated atherosclerotic plaque development, and this occurred in the absence of any changes in plasma lipids [102]. These studies reinforced a pro-atherogenic role for macrophage LPL. It is worth noting that although LPL has abundant expression in highly oxidative tissues, it is also detected in tissues like the kidneys, brain and macrophages where its binding properties are likely more important than its lipolytic activity [103]. Thus, the effect of macrophage LPL on atherosclerosis could be the result of its lipolytic activity and/or its ability to act as a receptor to assist remnant particle uptake. Related to the latter function of LPL, subsequent to lipolysis of lipoproteins, the remnant particles that detach from the endothelial GPIHBP1 platform have inactive LPL bound to them. It has been proposed that this inactive LPL may act as a hepatic receptor ligand to promote lipoprotein uptake [104–106]. When this process occurs in macrophages, LPL acted as a bridging molecule for remnant uptake to increase foam cell formation and lesion progression (Figure 1) [107].

### LPL in smooth muscle cells (SMC)

The above discussion brings forward the contribution of macrophages towards foam cell formation and development of atherosclerosis. However, more recent studies have also implicated SMCs in foam cell formation, at least in humans [108]. Thus, in mouse models of atherosclerosis, the lipid milieu along with the resident inflammation recruits’ monocytes across EC to initiate atherosclerosis [109]. In contrast, in human atherosclerosis, SMCs migration from the arterial media occurs before lipid accumulation and in fact SMC make up almost 50% of the foam cells in the plaque lesion [108]. It is possible that SMCs take up lipoprotein remnants through expression of scavenger receptors. However, LPL expression in SMC may also contribute to this mechanism. *In vitro*, SMC and macrophages synthesize LPL [110] which could act as a co-receptor to facilitate the binding of native and ox-LDL to HSPG (Figure 1) [111]. Interestingly, LPL has been detected in the fibrous cap of the atherosclerotic lesions [112]. Whether SMCs in the atherosclerotic lesion synthesize LPL *per se* or whether it is transferred from the macrophages is currently unknown. It should be noted that at least in the heart, the translocation of LPL has been reported from cells like the cardiomyocyte to endothelial cells [86]. In support, SMC interact with macrophages both directly and indirectly. For example, in the presence of macrophages, SMC increases their phagocytic activity by enhancing LPL and proteoglycans to promote lipoprotein uptake [113].

## LPL as a therapeutic target

Given the importance of LPL in FA delivery to multiple tissues in addition to its contribution to atherosclerotic plaque development, there are a number of pharmaceutical approaches that have been attempted to lower cardiovascular risk by targeting LPL. These include:

### Incretins

Oral glucose causes the release of gut hormones like glucagon-like peptide-1 (GLP-1) and glucose-dependent insulinotropic polypeptide (GIP) that amplify glucose-induced insulin secretion in addition to acting via multiple mechanisms to influence blood glucose [114]. Drugs that mimic GLP-1 (e.g., Semaglutide) and/or GIP (e.g., Tirzepatide) are gaining in popularity not only due to their control of blood glucose but also due to their ability to promote weight loss [115, 116]. Intriguingly, cardiovascular outcome trials demonstrate that long-term use of GLP-1 receptor agonists reduce cardiovascular complications of diabetes [117] or obesity [118] by mechanisms that not completely understood. It has been proposed that incretins could offer cardioprotection via their influence on lipid metabolism. In this regard, GLP-1 has been shown to regulate secretion of lipoproteins and cholesterol metabolism [119, 120]. GIP, on the other hand, demonstrated a regulatory role in lipid metabolism that occurred partially via LPL activation. Thus, in cultured 3T3-L1 cells and human adipocytes, GIP stimulated LPL activity in a dose-dependent manner [121–123]. These results were supported by *in vivo* studies which reported that GIP accelerated chylomicron TG clearance in dogs [124]. Similarly, human studies revealed that GIP infusion significantly increased LPL action, where LPL-derived FA largely contributed to an increase in re-esterification rate and TG storage in adipose tissue of lean individuals [125]. However, unlike adipose tissue, the effects of GIP on cardiac LPL level remain unclear. Given the opposing mechanisms of LPL regulation between adipose tissue and the heart, it is possible that GIP lowers cardiac LPL activity. Interestingly, eliminating GIP receptor signaling protected the heart against experimental myocardial infarction, and this was associated with reduced phosphorylation of HSL and increased cardiac TG storage [126]. As intramuscular TG accumulation is predominantly regulated by the action of HSL and LPL [127], the contribution of cardiac LPL to the altered lipid accumulation and the cardioprotective phenotype of Gipr−/− mice following myocardial infarction would be interesting to study.

### Apolipoprotein and angiopoietin-like protein inhibitors

Apolipoprotein C3 (APOC3) and angiopoietin-like protein (e.g. ANGPTL3) are known to inhibit LPL activity directly. Thus, a genetic reduction in *APOC3* increases LPL activity, reduces plasma TG and causes a decrease in coronary heart disease [128]. Related to targeting APOC3, two antisense oligonucleotides, Olezarsen and Volanesorsen, are currently in Phase 3 clinical trials to determine their therapeutic potential to lower circulating TG [129, 130]. Similar to APOC3, genetic variants in *ANGPTL3* impacts plasma TG [131]. As such, Evinacumab (human monoclonal antibody) is currently on the market to inhibit the action of APOC3 to lower TG levels [132].

### Omega-3 fatty acids (icosapent ethyl)

Used in statin-treated patients with elevated TG (≥150 mg/dL), who are at high risk of cardiovascular events due to established cardiovascular disease, or diabetes, and at least one other cardiovascular risk factor. Mechanism of action not completely understood and likely multifactorial and includes a decreased production and accelerated clearance of triglycerides [133]. It decreases VLDL synthesis and secretion by a) reducing hepatic lipogenesis (synthesis of FA from acetyl CoA), b) increasing beta-oxidation of FA and c) inhibiting TG-synthesizing enzymes (e.g., DGAT). It increases VLDL clearance by a) augmenting LPL activity directly or b) indirectly by reducing APOC3, an inhibitor of LPL. It’s a unique form of omega-3 fatty acid (eicosapentaenoic acid) that reduces VLDL-TG. Affects multiple atherosclerotic processes including endothelial function, oxidative stress, foam cell formation, inflammatory response (its anti-inflammatory), platelet aggregation and plaque rupture (it causes plaque regression) [134, 135].

### Fibric acid derivatives

Also called fibrates (e.g., fenofibrate and bezafibrate). Function primarily as peroxisome proliferator-activated receptors (PPAR) agonists (stimulates the PPARα receptor), thereby increasing the oxidation of fatty acids in liver (decreases VLDL production) and striated muscle (also kidney and heart). Also increases LPL activity (increased catabolism of circulating TGs increases the rate of clearance of TG) by both transcription upregulation of LPL and down regulation of APOC3 (an inhibitor of LPL) [136, 137].

### Statins

They are reversible, competitive inhibitors of HMG-CoA reductase. As a result, there is inhibition of intracellular cholesterol synthesis mainly in the liver. Because a precise amount of cholesterol is required in cells, on decrease of intracellular cholesterol, hepatocytes increase the expression of LDL receptors which then promote the extraction of LDL cholesterol from plasma secondarily. Are also known to influence LPL, especially in patients with Type 2 diabetes [138, 139]. These effects of statins occurred in a tissue specific manner, with an increased LPL production observed in skeletal muscle [140] and a decrease in LPL mass reported in macrophages [141].

## Discussion

The enzyme LPL is essential for circulating TG clearance, FA delivery for both oxidation and storage, and for prompting lipoprotein uptake by acting as a receptor. Given these multiple functions, changes in LPL would be expected to have diverse consequences. For example, a decrease in adipose tissue LPL would impede lipoprotein clearance resulting in augmented plasma lipids. In the heart, reduction in LPL, as observed with severe diabetes, causes a switch in substrate utilization to predominantly NEFA. This overwhelms the mitochondrial oxidative capacity leading to TG storage and lipid toxicity. However, depletion of macrophage LPL demonstrated beneficial effects against the development of atherosclerosis. Thus, when attempting to modulate LPL levels, one should consider the tissue and cell type in addition to the disease entity. In this regard, tissue or cell-specific manipulation of LPL offers promise to overcome the cardiac complications associated with obesity and diabetes.
